# Bat Species Diversity in the Merapoh Rich Limestone-rich Area within Lipis National Geopark, Malaysia

**DOI:** 10.3897/BDJ.12.e125875

**Published:** 2024-11-15

**Authors:** Aminuddin Baqi Hasrizal Fuad, Nur Zakirah Halmi, Hafiz M Yazid, Mohd Nur Arifuddin, Izereen Mukri, Siti Nurfarhana Zafirah Azidi, Jacqueline Clara Anak Chuat, Mohamad Iqbal bin Nurul Hafiz, Nur Nabilah binti A.Rahman, Khairun Nizam, Saberi Zoo, Fong Pooi Har, Suganthi Appalasamy, Jayaraj Vijaya Kumaran

**Affiliations:** 1 Faculty of Earth Science, Universiti Malaysia Kelantan, Jeli, Kelantan, Malaysia Faculty of Earth Science, Universiti Malaysia Kelantan Jeli, Kelantan Malaysia; 2 Malayan Rainforest Station, Kg. Gua Layang, Merapoh, Lipis, Pahang, Malaysia Malayan Rainforest Station, Kg. Gua Layang, Merapoh Lipis, Pahang Malaysia; 3 Persatuan Pemandu Pelancong Alam Semulajadi Taman Negara Pahang, Merapoh, Lipis, Pahang, Malaysia Persatuan Pemandu Pelancong Alam Semulajadi Taman Negara Pahang, Merapoh Lipis, Pahang Malaysia; 4 Lik Tin Environment Consultancy, Pt3445 Taman Desa Impian, Kg Sat, Tanah Merah, Kelantan, Malaysia Lik Tin Environment Consultancy, Pt3445 Taman Desa Impian, Kg Sat Tanah Merah, Kelantan Malaysia

**Keywords:** Merapoh, Lipis National Geopark caves, limestone karst, bat diversity

## Abstract

Merapoh, Pahang, is an area rich with limestone karst located within the Lipis National Geopark and home to the Sungai Relau gate of Taman Negara Pahang, a totally protected rainforest in Malaysia. Much of the research conducted here is mainly inside the National Park, with few published faunal records for the Merapoh caves. This study compiled the data on the bat species diversity of eight Merapoh caves (March 2020 to March 2022) using mist nets and harp traps. Our results indicate that Chiroptera diversity at Merapoh caves is rich, with a total of 32 species recorded from 865 individuals and four new locality records for the State of Pahang, namely *Rousettusleschenaultii*, *Lyrodermalyra*, *Rhinolophuscoelophyllus* and *Hipposiderospomona*. Gua Gunting has the highest diversity of bats recorded in this study (19 species). Significant Merapoh caves with bat colony roosts in Merapoh include Gua Jinjang Pelamin (*Eonycterisspelaea* & *Rousettusleschenaultii*), Gua Tahi Bintang (*Hipposideroslarvatus*) and Gua Pasir Besar (*Miniopterusmedius*). *Rhinolophusconvexus*, previously recorded only in upper montane rainforests, was also recorded in Merapoh caves indicating that this species can also be found in lower elevations than previously thought. Based on the findings of the current study and additional records from two previous studies, the Merapoh bat species diversity checklist totalled up to 38 species. On the whole, the rich bat diversity in Merapoh is reflective of its immense limestone karst landscape, which highlights the reason Lipis National Geopark has been recently gazetted. Future bat research should continue here and in other karsts within Lipis Geopark to sustainably conserve biological diversity, manage geological structures and raise awareness amongst the locals to appreciate their national heritage.

## Introduction

Lipis National Geopark in the State of Pahang is a newly-gazetted Geopark in Malaysia (November 2023) with an area of 5,198 km^2^ showcasing 28 national geological heritage, six biologically diverse areas and 18 cultural heritage sites (Lau and Lau 2023). The Merapoh area within Lipis Geopark is surrounded by several forest reserves and borders Taman Negara Pahang, the largest national park in Peninsular Malaysia. The main geological feature in Merapoh is the large number of limestone hills from the Gua Musang formation, which were formed around the Permian-Triassic period millions of years ago (Jeyanny et al. 2019). The abundance of limestone hills and forested regions have brought forth the richness in the biodiversity of the Merapoh area, particularly karst flora and fauna

Bats belonging to the order Chiroptera, are the only mammals with the capability of sustained flight. The order Chiroptera is a diverse order encompassing more than 1,300 species globally that can be traditionally divided into Megachiroptera and Microchiroptera (Srinivasulu et al. 2010, Lim and Wilson 2019). However, recent studies suggested that these suborders could be substituted with new suborders named Yinpterochiroptera and Yangochiroptera (Teeling et al. 2005, Lei and Dong 2016). Bats are particularly diverse in the Tropics, accounting for around 40% of the mammal species in Southeast Asia (Lim and Wilson 2019). There are about 143 bat species in Malaysia, 113 of them being found in Peninsular Malaysia (Lim et al. 2016, Lim et al. 2017, Juliana Senawi and Norhayati Ahmad 2021).

The limestone karst landscape, where caves are prominent, provides permanent roosting sites for bats and foraging resources in the forest above and surrounding the limestone hill (Struebig et al. 2008, Furey et al. 2010). Furthermore, limestone karst landscapes are biodiversity hotspots that support a rich diversity of flora and fauna with a high endemism rate which covers about 10% of the total land area in Southeast Asia, highlighting the biological importance of this landscape (Tolentino et al. 2020, Liew et al. 2021). Limestone karst landscape typically face threats from limestone quarrying and habitat degradation, as limestone hills have no direct legal protection status in Malaysia unless the landscape is in a protected area such as Gua Niah in Niah National Park, Gua Gomantong in Gomantong Forest Reserve and also Gua Gajah inside Taman Negara Pahang Sungai Relau (Day 2011, Tolentino et al. 2020). While not every limestone karst can feasibly be included within protected areas, geoparks are the next best thing, as the status provides a form of legal protection to karst areas by empowering local communities working with government agencies. The National Geopark initiative in Malaysia aims to holistically manage the nation's geological resources intertwined with biological diversity and cultural heritage for a sustainable economic output. This bottom-up approach will hopefully instil a sense of pride amongst locals to conserve their natural heritages, along with the opportunity to be recognised internationally through UNESCO Global Geopark designation (UNESCO 2015,Lipis Geopark 2023).

Though the many geological and fossil research published in the Lipis District contribute greatly to the Geopark gazettement, there have been few faunal studies here. Merapoh, in particular, has many studies that mainly conducted research inside Taman Negara Sungai Relau in contrast to the limestone hills and forested areas outside the said National Park (Nizam et al. 2006, Kawanishi and E. Sunquist 2008, Jeyanny et al. 2019). Despite the significant research potential of karst landscapes, studies on bat diversity in Merapoh have been scarce, even though bats are strongly associated with caves. Thus, it is imperative that this gap of knowledge is addressed as Merapoh serves as a transitional area between protected rainforest habitat and human-modified habitat in the larger scope of Lipis Geopark. Last but not least, this study aims to find and compile the bat species composition of Merapoh caves. We also hypothesised that the cave that has been dominated by one bat species will have lower species richness overall. The outcome of this study will provide a picture of the overall bat diversity in this rich limestone karst region and be useful for the holistic management of bat conservation and limestone hill resources.

## Study site description

**Figure 1.**
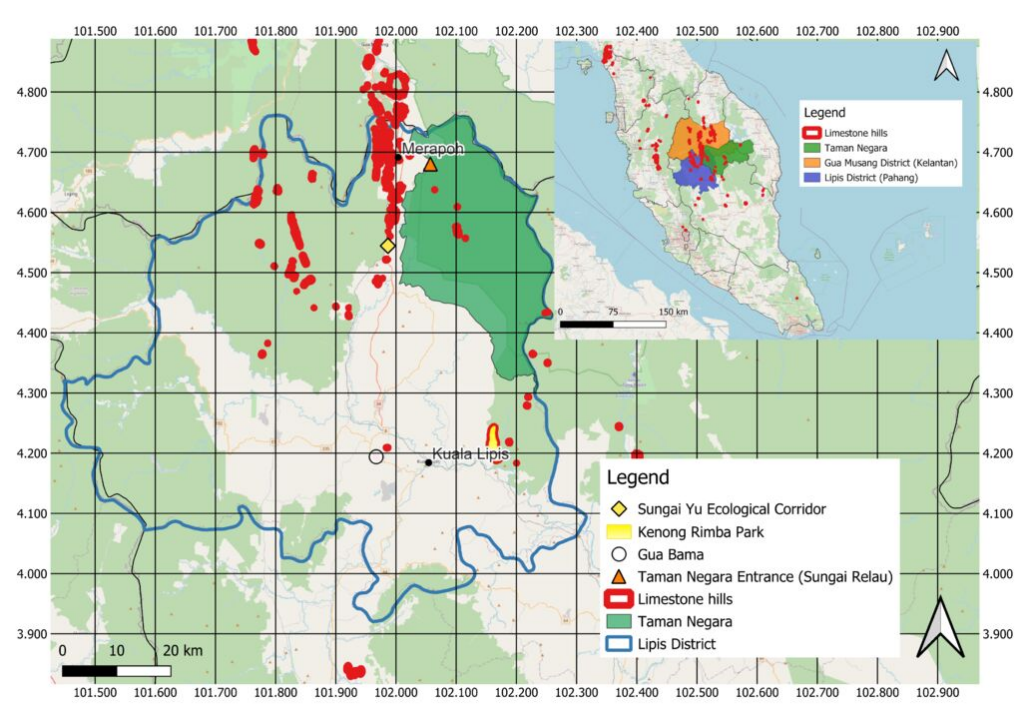
**Figure 1.** The location of Lipis National Geopark in Peninsular Malaysia and some notable locations inside Lipis. Kuala Lipis is the district capital of Lipis. Merapoh is located in northern Lipis where our study took place. Gua Bama and Kenong Rimba Park are some of the prominent geological features in Lipis Geopark. Sungai Yu Ecological Corridor is part of the Central Forest Spine that connects Taman Negara with Tanum Forest Reserve and Sungai Yu Forest Reserve which overall links to other forested areas in the region (Meisery et al. 2020).

As its name implied, Lipis National Geopark encompasses the entire Lipis District with geological formations (Bentong-Raub suture zone and Gua Bama Permian-Triassic boundary), limestone karst landscapes (Merapoh and Kenong) since the Ordovician period, plus residence to a large portion of Taman Negara Pahang, one of the world’s oldest rainforests (Lau and Lau 2023). In northern Lipis, there lies Merapoh, a small town that is located next to the Pahang-Kelantan border about 30 km from Gua Musang in Kelantan and 80.7 km from Kuala Lipis in Pahang. This town is home to the lesser-known Sungai Relau gate of Taman Negara Pahang and is surrounded by a large number of limestone hills forming the Merapoh cave system which consists of 85 explored caves believed to be 130 million years old, along with many more unexplored caves waiting to be discovered. Fieldwork sampling of bats was carried out at eight caves in Merapoh: Gua Air Mata Dayang, Gua Persit, Gua Gunting, Gua Jinjang Pelamin, Gua Kalong, Gua Katak, Gua Pasir Besar and Gua Tahi Bintang. Fig. 1 shows the map of Lipis District of Pahang, Malaysia which represents the recently-gazzetted Lipis National Geopark.

Gua Katak, Gua Persit and Gua Tahi Bintang are located in an area that has a considerable amount of local agriculture activities, such as oil palm plantations, mixed fruit orchards and rubber plantations. Gua Air Mata Dayang is literally behind a villager’s house and his durian orchard, while Gua Kalong is surrounded by small forest patches that have been gradually opened up for agricultural purposes.

**Figure 2.**
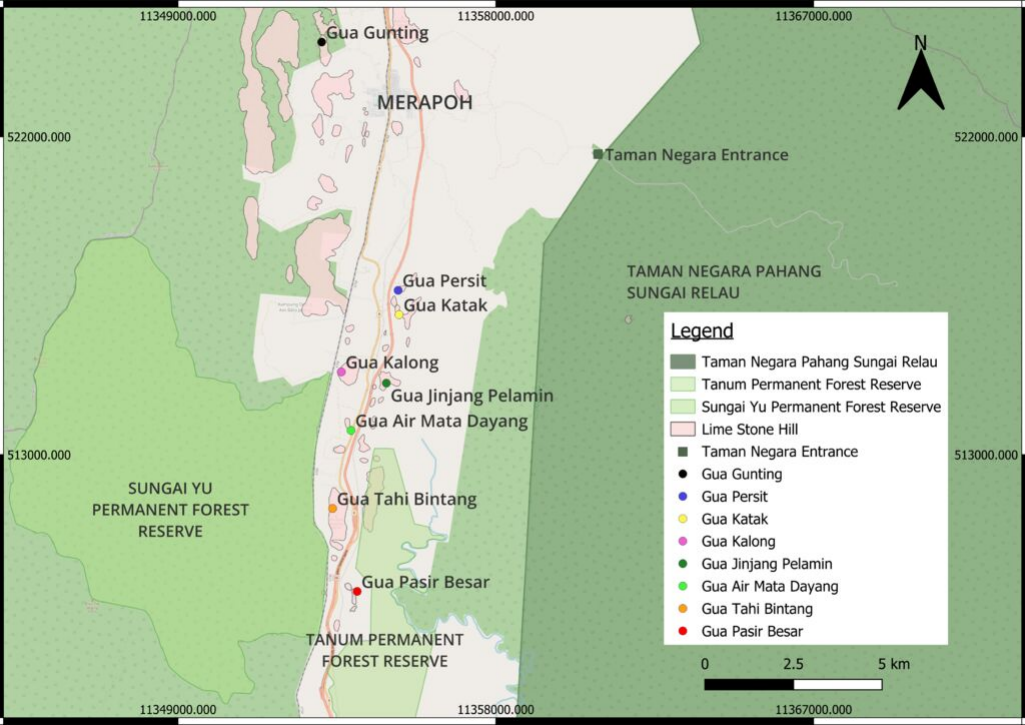
The location of eight sampled caves in Merapoh (Limestone hills map layer, based on Liew et al. (2021)). Northmost of Merapoh is Gua Gunting. Gua Persit and Gua Katak are located in the middle of the map, south of Gua Gunting. Continuing south, Gua Kalong and Gua Jinjang Pelamin are on the opposite side of the major road. The southernmost cave is Gua Pasir Besar, which is at the end of Merapoh village and near the Sungai Yu Ecological Corridor. North of Gua Pasir Besar is Gua Tahi Bintang and Gua Air Mata Dayang, which are two of the main caves opened for caving tourism.

Three caves (Gua Gunting, Gua Jinjang Pelamin and Gua Pasir Besar) have substantial forest areas in their vicinity. Gua Gunting, for instance, is located in a sizable forest patch that is connected to the Persit Forest Reserve. Gua Jinjang Pelamin has scattered forest patches that also link to Sungai Relau, which borders Taman Negara Pahang Sungai Relau, a protected rainforest area. Lastly, Gua Pasir Besar is sandwiched between two forest reserves, Tanum Forest Reserve and Sungai Yu Forest Reserve which are connected to the Taman Negara region. Fig. 2 shows the map of all eight sampled caves in Merapoh, Pahang.

## Materials and Methods

The sampling period is between March 2020 to March 2022 for a total of eight caves. All eight caves have varying total bat sampling nights as samplings were conducted opportunistically, but each cave does have a minimum of four trapping nights. The total sampling effort for all eight Merapoh caves is 156 trapping stations (78 harp traps and 78 mist nets) in 39 trapping nights. Table 1 lists all the GPS locations of sampled Merapoh caves, sampling dates and number of trapping nights of all caves.

Two mist nets (9 m x 4 m, mesh size: 4 cm) and two harp traps (4 bank, 2 m^2^ metal frame) were used for each sampling night at all eight caves to capture bats at two locations: 1) inside the cave or near the cave entrance and 2) at the forest trail where bats are likely to forage or potential bat flyways. All traps were set up 5 to 100 metres away from each other, depending on the conditions of the trap placement area within an approximate 250 m radius from the main cave entrance. The traps were opened at 18:30 hours, checked every hour starting 20:00 hours and closed at 23:00 hours. The bats captured in both mist nets and harp traps were transferred into cloth bags, one for each individual, to be processed for species identification. All standard body measurements (forearm length, tibia length, ear length and body weight), gender and maturity state of each captured bat were recorded. The body measurements and the bat’s physical features were used for species identification referring to the identification keys from Kingston et al. (2009) and Francis (2019). The bats that have been measured and identified were released back near their captured cave area on the same night. The type of traps used and the handling of the captured bats were conducted ethically following the guidelines from the American Society of Mammalogists (Sikes and the Animal Care and Use Committee of the American Society of Mammalogists 2016).

### Statistical analysis

The Merapoh bat species checklist is compiled from the bat sampling results of the eight caves. Comparisons between the sampled caves were made for the following aspects: diversity and abundance of bat species, dominant bat species in each cave and bat species distribution between the caves using the Shannon-Wiener index, species equitability and dominant species index (Magurran 2004). A rarefaction curve was constructed for standardisation as the bat sampling efforts (number of sampling nights) for each cave are different. To determine and visualise the variation in bat assemblages amongst the eight sampled caves, the non-metric multidimensional scaling (NMDS), based on Bray-Curtis dissimilarity index, was calculated (Magurran 2004). All analyses were conducted using PAST software (Hammer et al. 2001). Lastly, a comparison with previous bat diversity studies done in Merapoh caves were prepared to generate the full bat species composition in Merapoh, with their conservation status obtained from the global IUCN Red List (IUCN 2023) and the Red List of Mammals for Peninsular Malaysia Version 2.0 (PERHILITAN 2017).

## Results

During the sampling period (March 2020-March 2022), a total of 865 individuals consisting of 32 bat species from seven families were captured at eight caves in Merapoh. For the fruit bat species complex, *Cynopterusbrachyotis*, the two forms (‘Forest’ and ‘Sunda’) were highlighted due to the capture of different body sizes and the occurrence of different habitat types that can be utilised by *C.brachyotis* ‘Forest’ and *C.brachyotis* ‘Sunda’ (Abdullah and Jayaraj 2006). The species identification of these two forms relied on body sizes and calculations from a predictive model proposed by Jayaraj et al. (2012).

The most abundant bat species caught is *Hipposideroskunzi* (n = 170), followed by *Hipposideroslarvatus* (n = 117) and *Eonycterisspelaea* (n = 100). By comparison, *Rhinolophuscoelophyllus*, *Rhinolophusconvexus*, *Hipposideroslylei* and *Hipposiderospomona* were recorded as singleton captures. Gua Gunting has the highest number of bat captures (n = 205), while Gua Persit is the cave with the lowest bat capture numbers (n = 15). Table 2 shows the diversity and abundance of bat species in all the sampled caves. The occurrence data of all bats captured in all eight caves in this study can be viewed in Suppl. material 1.

Amongst the 32 bat species recorded, *Rhinolophuspusillus* (n = 40) and *Hipposideroskunzi* (n = 170) were found in seven out of eight caves sampled, while *Cynopterushorsfieldii* (n = 14) and *Rhinolophusstheno* (n = 76) were found in six caves. Furthermore, *Rhinolophusaffinis* (n = 48), *Hipposideroscineraceus* (n = 25) and *Hipposideroslarvatus* (n = 117) were recorded in five different caves. As for the rarest bat species amongst the eight caves, a total of nine species (*Rhinolophuscoelophyllus*, *R.convexus*, *Hipposiderosdiadema*, *H.lylei*, *H.pomona*, *Kerivoulapellucida*, *Murinasuilla*, *Miniopterusmagnater* and *Myotisater*) were found at their respective cave area only.

On the surface, Gua Gunting looks to be the most diverse cave in Merapoh in our study, with a total of 19 bat species (eight sampling nights, n = 205) recorded, while Gua Persit is the least diverse at five species (four sampling nights, n = 15). However, the indices show a different story with Gua Jinjang Pelamin being actually more diverse (H’ = 2.24) than Gua Gunting (H’ = 1.68). For the least diverse cave, it is actually Gua Kalong (H’=0.67) even with the higher number of individuals and total species recorded compared to Gua Persit (H' = 1.03). Other caves showed high species richness, but have low dominance like Gua Gunting (D' = 0.274), Gua Katak (H’ = 1.95, D’ = 0.18) and Gua Pasir Besar (H’ = 1.78, D’ = 0.21). Gua Jinjang Pelamin also has the highest species evenness index (J’ = 0.971) although the cave has the highest number of total captures for this species (n = 94) in this study and is known by the locals to harbour the largest colony of *Eonycterisspelaea* in Merapoh.

On the other hand, Gua Kalong has the highest dominance index (D’ = 0.701) with *Hipposideroskunzi* as the dominant bat species. *Hipposideroskunzi* is also the dominant bat species for two other caves which are Gua Katak and Gua Gunting (shared with *Hipposiderosdyacorum*). *Eonycterisspelaea* is the dominant bat species in Gua Jinjang Pelamin and Gua Persit, while *Hipposideroslarvatus* is the dominant bat species in Gua Tahi Bintang and Gua Air Mata Dayang, though their species dominance differ in each cave; highly dominant in Gua Tahi Bintang (D’ = 0.56), while low dominance at Gua Air Mata Dayang (D’ = 0.28).

**Figure 3.**
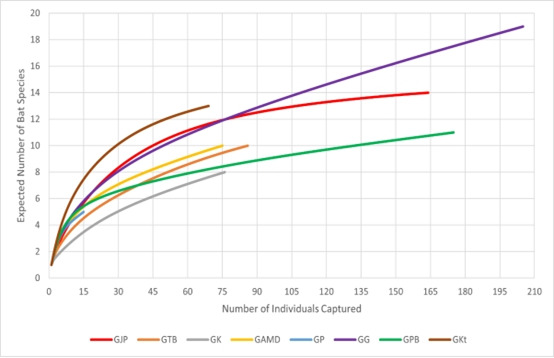
Rarefaction curve showing the number of bat species in eight caves in Merapoh. Abbreviations: **GJP**: Gua Jinjang Pelamin, **GTB**: Gua Tahi Bintang, **GK**: Gua Kalong, **GAMD**: Gua Air Mata Dayang, **GP**: Gua Persit, **GG**: Gua Gunting, **GPB**: Gua Pasir Besar, **GKt**: Gua Katak.

As the bat sampling conducted in Merapoh for this study is opportunistic, we do not have the same number of sampling nights for each cave. By extrapolating a rarefaction curve (Fig. 3) with a standardised number of individual captures (n = 15), Gua Katak is surprisingly the cave with the highest expected number of species, with seven compared to Gua Gunting, with only five expected bat species. Similarly, Gua Persit is not the cave with the lowest expected number of species, but Gua Kalong with three expected bat species.

**Figure 4.**
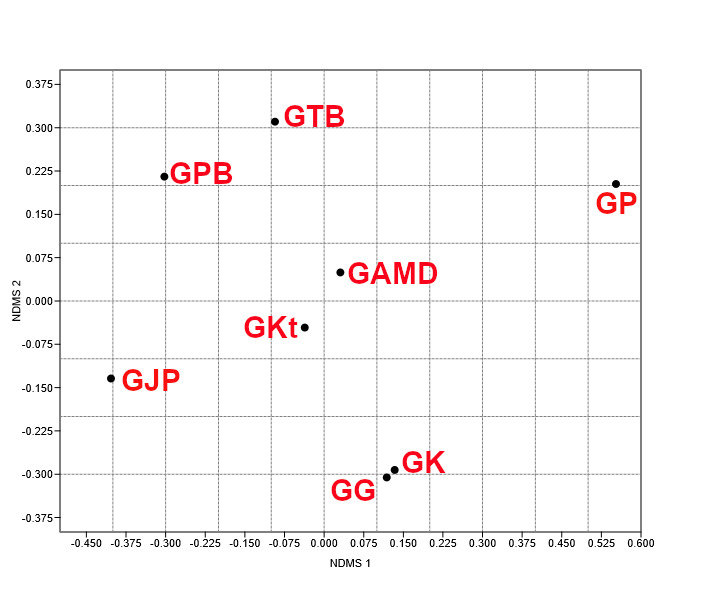
Non-metric multidimensional scaling (NMDS) ordination of bat assemblages at eight sampled caves in Merapoh within the Lipis National Geopark, Pahang, Malaysia. Keys: **GJP**: Gua Jinjang Pelamin, **GTB**: Gua Tahi Bintang, **GK**: Gua Kalong, **GAMD**: Gua Air Mata Dayang, **GP**: Gua Persit, **GG**: Gua Gunting, **GPB**: Gua Pasir Besar, **GKt**: Gua Katak.

From the NMDS ordination (stress = 0.159), Gua Kalong and Gua Gunting are closely grouped together with both caves having six overlapping bat species. Four caves are loosely grouped together in pairs; 1) Gua Air Mata Dayang and Gua Katak, 2) Gua Tahi Bintang and Gua Pasir Besar. Gua Jinjang Pelamin and Gua Persit are on the opposite side of the NMDS graph although both caves recorded similar fruit bat species (*Eonycterisspelaea* and *Cynopterushorsfieldii*). The NMDS based on Bray-Curtis dissimilarity index can be seen in Fig. 4.

Based on Table 3, the full bat species composition in Merapoh increased to 38 species in total. *Hipposiderospomona* is listed as Endangered globally (IUCN 2023), but Data Deficient for Peninsular Malaysia, while *R.convexus* is listed as Data Deficient for both conservation statuses. Other bat species that are listed as Data Deficient in the Red List of Mammals in Peninsular Malaysia, but Least Concern in IUCN 2023, are *Macroglossusminimus*, *Emballonuramonticola*, *Lyrodermalyra*, *Rhinolophuscoelophyllus*, *Hipposiderosdyacorum*, *H.lylei*, *Hesperoptenusblanfordi* and *Myotisater*. *Eonycterisspelaea* is listed as Near Threatened, while *Hipposideroskunzi* is listed as Vulnerable for Peninsular Malaysia. For Near Threatened status in the global IUCN 2023, the species listed include *Rousettusleschenaultii*, *Nycteristragata* and *Kerivoulapellucida*, but both *N.tragata* and *K.pellucida* are only Least Concern in the Red List of Mammals in Peninsular Malaysia.

Furthermore, this bat species checklist updates four new locality records for Pahang: *Rousettusleschenaultii*, *Rhinolophuscoelophyllus*, *Lyrodermalyra* and *Hipposiderospomona*. One intriguing capture for this study is *Rhinolophusconvexus*, which has only been found in the upper montane rainforest. The limestone hills in Merapoh where this bat was captured, do not reach the previously observed elevation (1,600 m) for this bat species. Given the Data Deficient status of this species, further genetic studies will be needed to determine whether their elevation limit can be expanded towards limestone hill forests or whether this *R.convexus* record in Merapoh represents a distinct bat species (Francis 2019).

### Species accounts

#### Family Pteropodidae

***Rousettusleschenaultii* Desmarest, 1820 (Leschenault's Rousette)**

**Figure 5.**
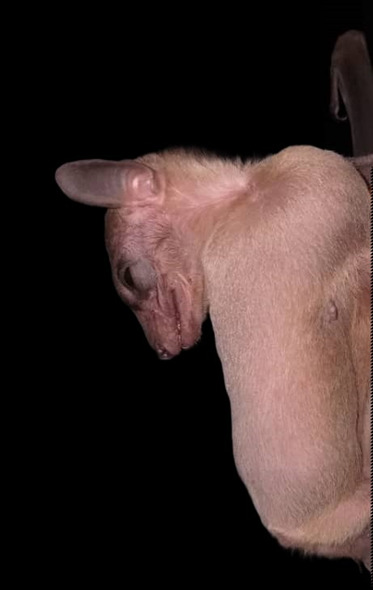
A Leschenault's Rousette (*Rousettusleschenaultii*) individual sampled in this study.

This species is a new locality record for the State of Pahang. A total of 21 individuals were captured in Merapoh in which 19 were from Gua Jinjang Pelamin and two were from Gua Tahi Bintang. This species is one of the few fruit bat species that roost inside caves and can be found roosting with *Eonycterisspelaea* in Gua Jinjang Pelamin for this study and also Batu Caves, Selangor (Francis 2019, Nuratiqah et al. 2023). *Rousettusleschenaultii* can use a rudimentary echolocation call by tongue clicking (Raghuram et al. 2007). Its diet is mainly fruits, but will opportunistically exploit nectar and pollen when floral resources are plentiful (Stewart and Dudash 2016). Fig. 5 shows an individual of this species caught during sampling.

#### Family Megadermatidae

***Lyrodermalyra* E. Geoffroy, 1810 (Greater False Vampire Bat)**

**Figure 6.**
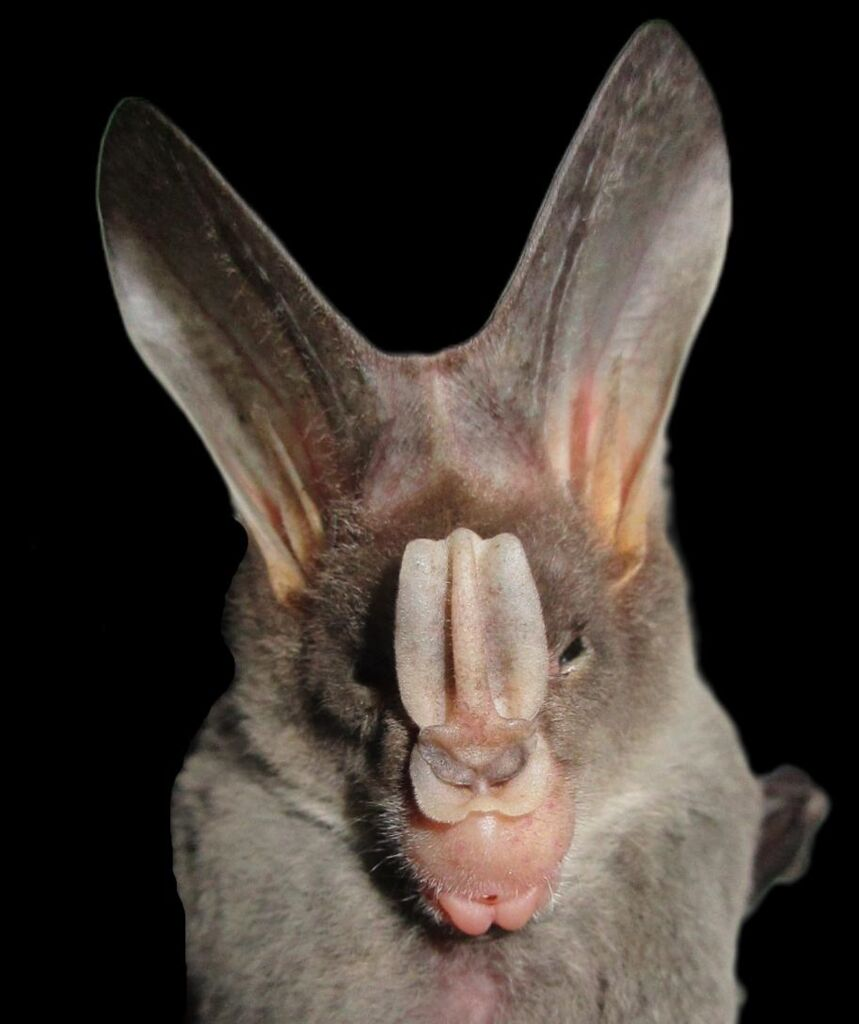
A Greater False Vampire Bat (*Lyrodermalyra*) individual.

This species is a new locality record for the State of Pahang. Three individuals were captured in Merapoh with two being at Gua Kalong and one at Gua Tahi Bintang. *Lyrodermalyra* has been recorded in Peninsular Malaysia within two States; Perak and Selangor (Lim et al. 2017). This bat species has been found roosting in small numbers in caves, abandoned buildings and tunnels (Francis 2019). This species is a seasonal predominantly gleaning carnivore bat that preys on vertebrates when insect prey resources are scarce; vertebrate prey includes lizards, small mammals and birds (Gual‐Suárez and Medellín 2021). The smaller relative, *Megadermaspasma* differs from *L.lyra* by having a shorter posterior noseleaf, a heart-shaped intermediate noseleaf and its diet consists mainly of large insects with a lesser frequency of hunting small vertebrates (Francis 2019, Gual‐Suárez and Medellín 2021). Fig. 6 shows a Greater False Vampire Bat sampled during this study.

#### Family Rhinolophidae

***Rhinolophusconvexus* Csorba, 1997 (Convex Horseshoe Bat)**

One individual was captured at Gua Gunting (elevation: 191 m). *Rhinolophusconvexus* is a species that has very little information and is listed as Data Deficient (DD) in the IUCN Red List of Threatened Species and the Red List of Mammals in Peninsular Malaysia. This bat species has been recorded in Cameron Highlands, Pahang in Peninsular Malaysia (Csorba 1997). A study in 2020 has also since recorded the existence of *R.convexus* in the State of Terengganu (Nurulhuda et al. 2020). The specimens recorded were obtained in Sungai Buweh, Kenyir area (elevation: 204 m) which is close to our Gua Gunting elevation in Merapoh, but this elevation aspect of *R.convexus* was not highlighted by the previous researcher (Nurulhuda et al. 2020). The current distribution of this species is very limited with specimens only known from Peninsular Malaysia and possibly Laos, though further taxonomic work is needed to verify whether the Laos specimens indeed represent *R.convexus* (Csorba et al. 2016).

**Figure 7.**
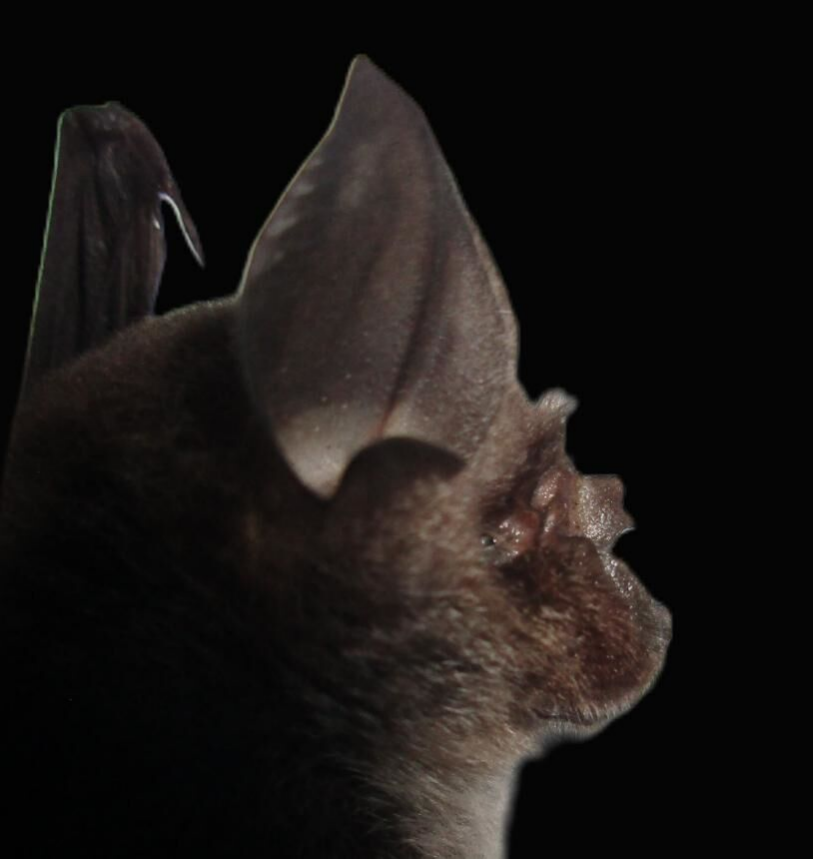
A Convex Horseshoe Bat (*Rhinolophusconvexus*) individual captured during sampling.

This species was first described in Cameron Highland, Pahang, and has been found in upper montane rainforest (elevation: 1600 m) in Peninsular Malaysia (Csorba 1997, Csorba et al. 2016). The specimen captured in Merapoh indicates a new elevation record for *R.convexus*. This finding suggests a possibility for these bats to traverse from the montane forest region to lower-elevation hill forests as there is no strong indication that this bat species exclusively roosts in montane forests (high-elevation habitats). Since very few specimens have ever been captured, the habitat, ecology, population and distribution information for this bat species is still uncertain. A photograph of a *R.convexus* is shown in Fig. 7.

#### *Rhinolophuscoelophyllus* Peters, 1867 (Croslet Horseshoe Bat)

**Figure 8.**
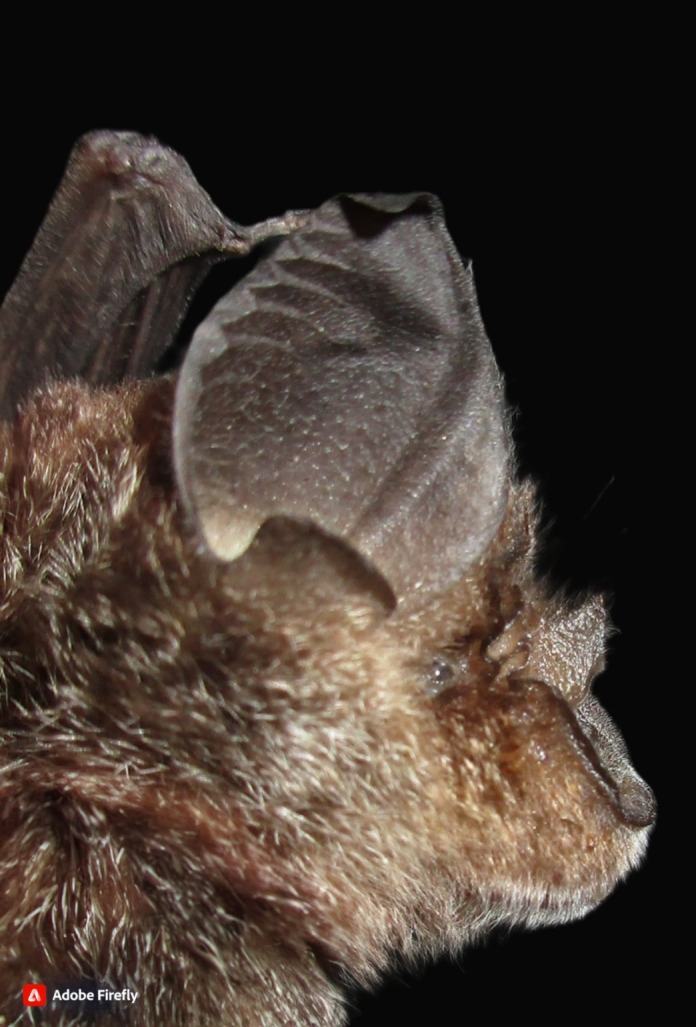
A Croslet Horseshoe Bat (*Rhinolophuscoelophyllus*) individual captured in this study.

One individual was captured at Gua Gunting, with this bat species being a new locality record for Pahang. *Rhinolophuscoelophyllus* has been recorded in Peninsular Malaysia within three States; Kedah, Perlis and Selangor (Lim et al. 2017). This species can be confused with a similar-looking species, *R.shameli* which has a broader noseleaf, generally lower echolocation call frequency and is not found in Peninsular Malaysia (Francis 2019, Furey et al. 2020). *R.coelophyllus* roosts in limestone caves and have been found foraging in various forests including lowland forest to hilly forests (Francis 2019). An individual of this species can be seen in Fig. 8.

#### Family Hipposideridae

***Hipposiderospomona* K. Andersen, 1918 (Large Eared Roundleaf Bat)**

One individual of *Hipposiderospomona* was caught at Gua Gunting with this species being a new locality record for the State of Pahang. *Hipposiderospomona* has been recorded in Peninsular Malaysia within four States; Perlis, Perak, Kelantan and Melaka (Lim et al. 2017). As its name suggested, *Hipposiderospomona* have very large, rounded ears amongst the small Hipposiderid bats. This bat species mainly roosts inside caves and can be found foraging in disturbed areas aside from forests (Francis 2019).

**Figure 9.**
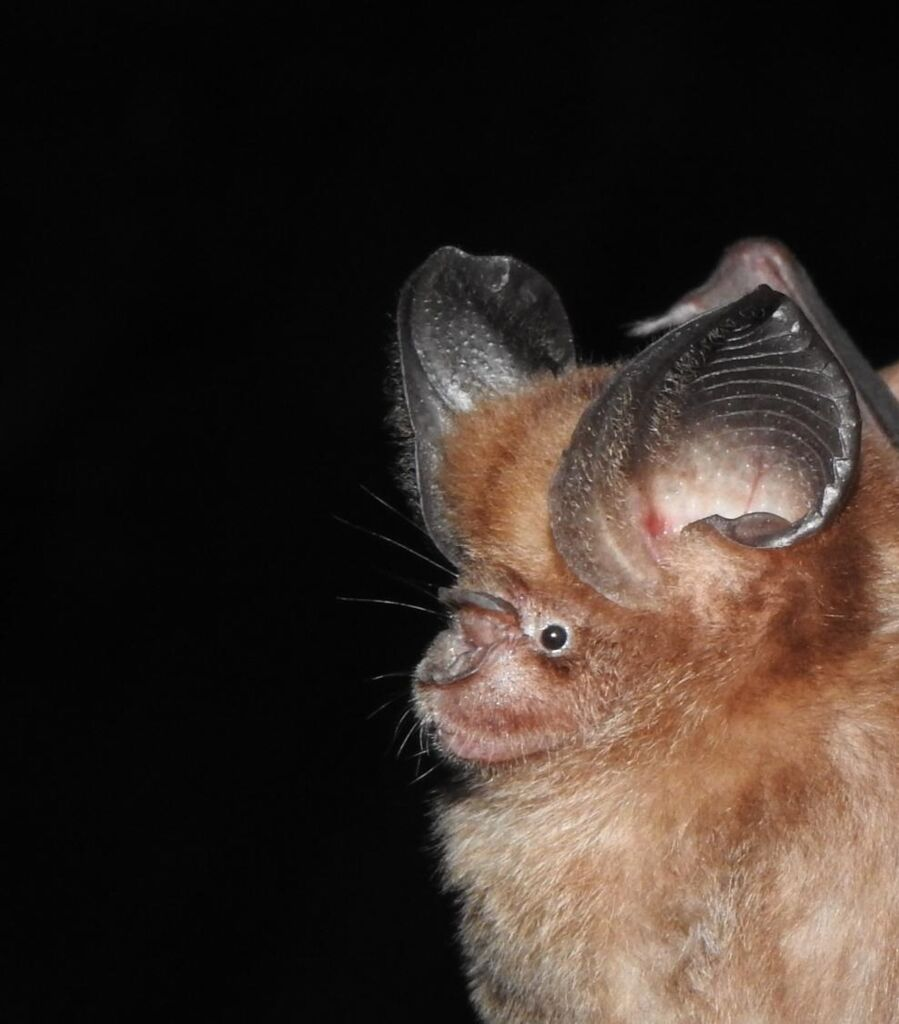
A Large Eared Roundleaf Bat (*Hipposiderospomona*) individual.

*Hipposiderospomona* have unresolved taxonomy due to the lack of DNA barcodes from Peninsular Malaysia and genetic analyses have yet to fully set the boundaries, particularly the uncertainties with a complex of species with the populations of *H.pomona* from Peninsular Malaysia, the rest of mainland Southeast Asia countries and southern China (three subspecies) are still disjunct (Douangboubpha et al. 2010, Lim et al. 2017). The *H.pomona* from Peninsular Malaysia could possibly represent a distinct taxon either species or subspecies (Douangboubpha et al. 2010; Murray et al. 2018). The specimen captured in Merapoh will hopefully contribute for future taxonomic research for this species complex. Fig. 9 shows a photagraph of an individual of this species.

## Discussion

Firstly, it is not at all surprising that bat species diversity in Merapoh area is high as the number of limestone hills containing many cave structures allows bats to utilise this area as roosts. Bats are often associated with caves and limestone karst ecosystems in general, where bats functions as primary contributors to the organic resources and nutrient flow inside the caves for other cave fauna such as small invertebrates (spiders, earwigs and centipedes), cave fish, frogs and microbes (Clements et al. 2006, Sakoui et al. 2020). Other limestone karst ecosystems also harbour many bat species such as Taman Negara Gunung Mulu, Malaysia that is known to harbour 41 species of bats. The Mulu cave system is also home to millions of *Chaerephonplicatus* occupying a single cave (Deer Cave). The Sangkulirang limestone karst formations in Kalimantan, Indonesia has 36 bat species, while the Kim Hy Nature Reserve in Vietnam is known to harbour 36 bat species (Suyanto and Struebig 2007, Furey et al. 2010, Isham Azhar et al. 2013, Tolentino et al. 2020).

Our findings in Merapoh indicate that several caves are deemed significant in bat diversity conservation. Starting with Gua Jinjang Pelamin, the cave houses the primary colony of *Eonycterisspelaea* and a refuge for *Rousettusleschenaultii* (n = 19) in which both bat species are important pollinators of durian, providing their pollination services to the less-intensively managed durian orchards in the surrounding area (Aminuddin Baqi et al. 2022). Other caves include Gua Tahi Bintang, which has a a large colony of *Hipposideroslarvatus* and Gua Pasir Besar, home to a large community of *Miniopterusmedius* and possibly other *Miniopterus* sp. Although it was possible that these species can occupy other sampled caves, the habitat quality and cave structure complexity, to a certain extent, may influence the distribution and population of such species of bats (Azhar et al. 2015, Phelps et al. 2016, Barros et al. 2020). Habitat quality degradation reduces the abundance and diversity of food resources (fruits and insects) as some bat species prefer certain food resources, like how fruit bats (*Cynopterusbrachyotis* and *E.spelaea*) forage more in low crop density plantations and insectivorous bats on less intensive farms with minimal agrochemical use (Wickramasinghe et al. 2003, Azhar et al. 2015, Syafiq et al. 2016, Wagner et al. 2021).

The results from our study partially indicate that caves with greater dominance have lower species diversity, as is the case for Gua Kalong and Gua Tahi Bintang. Gua Tahi Bintang is also another significant cave with the largest colony of *Hipposideroslarvatus* that can easily be seen here in Merapoh compared to other caves, suitable for long-term monitoring and education for this bat species. Nonetheless, Gua Jinjang Pelamin did not follow our hypothesis. This cave exhibited the highest species richness and highest species evenness amongst the eight sampled caves, despite *Eonycterisspelaea* being its dominant species, which accounted for 57.32% of the total individuals captured here. This unexpected result suggests that there are possibly other factors affecting the dominance of a bat species and the diversity of bat species inside and surrounding the caves. Hence, we should not make the assumption that just because a cave has a primary colony of one bat species, the bat diversity of the cave is low.

Furthermore, cave structure complexity does play a role in bat species selecting roosting sites inside caves as a large cave can support a larger number of bat populations and a complex cave with many chambers indirectly influences the microclimate of a bat colony's roosting site (Cigna 2004, Glover and Altringham 2008). In spite of these cave features, cave selection by bats is also significantly influenced by the habitat quality surrounding the cave. Many cave areas experience high anthropogenic activities, usually involving habitat destruction and fragmentation (Struebig et al. 2009, Phelps et al. 2016). The caves in Merapoh are fairly fragmented, with agricultural areas and secondary forest patches in between. Gua Persit is an extreme example of the isolation of limestone karst outcrops and caves in Merapoh, where the cave is surrounded by oil palm plantations and a major highway nearby (approximate distance to nearest forested area is 6.5 km). According to the villagers here, Gua Persit used to be a guano-collecting hotspot for the village, but nowadays, there are few guano sediments. The isolation of Gua Persit increases the foraging distance for bats, which may cause a long term reduction in bat population size inside Gua Persit, either due to an actual population decrease or migration of the bat colony to another cave. The bat population decline by indirect isolation of limestone karsts situation is closely similar to the conclusion brought up in another karst landscape by Struebig et al. (2009), though further research would need to be conducted to verify our conjecture here in Gua Persit at Merapoh. Limestone karst outcrops are technically land ‘islands’ in which the degree of isolation is amplified by habitat fragmentation (Clements et al. 2006). While insectivorous bats roosting inside caves are less impacted by limestone karst fragmented landscape than foliage-roosting bats, the overall bat diversity may erode, negatively affected by ‘island’ isolation and degradation particularly the forest foragers bat species (Hazard et al. 2023). The overall diversity and abundance of bats may also decrease and experience changes in composition due to biotic relaxation. Gua Air Mata Dayang, Gua Tahi Bintang, Gua Katak and Gua Kalong have a lesser degree of isolation as these caves still have forest patches and are surrounded by mixed fruit orchards or rubber plantations, which do support a satisfactory level of insect abundance. Gua Gunting and Gua Pasir Besar are positioned at a more favourable location despite habitat fragmentation, as these caves not only have forest patches (0.2-1 km^2^), but are also near a forest reserve border (less than 5 km) and are connected to Taman Negara, respectively.

When comparing this study bat diversity results with two other past studies in Merapoh as shown in Table 3 (Ratnam et al. 1989, Muhammad Aminuddin Baqi et al. 2020), the Merapoh bat diversity count increased to 38 bat species. Most of these bat species are recorded outside of Taman Negara Pahang Sungai Relau, except for Ratnam et al. (1989) whose team did some bat sampling using mist nets inside Taman Negara and a banana plantation near a Merapoh limestone hill. The high bat diversity count shows that limestone karsts are biodiversity arks and Merapoh should also be acknowledged as one of Peninsular Malaysia’s priority regions for bat conservation (Clements et al. 2006, Suyanto and Struebig 2007). The recent gazettement of Lipis Geopark area as a prime limestone geopark is a step in the right direction for bat conservation in Pahang.

The significance of Merapoh Caves and at a larger scale the Lipis Geopark cannot be understated for bat conservation, with many cave-roosting bat species and eight fruit bat species being found in this district. The current bat diversity shown in this study is an understatement of bat diversity in the area as there are still many limestone hills and caves in Merapoh that have yet to be sampled. This sampled study was only able to survey bats in eight Merapoh caves out of the 85 known caves here. Further bat samplings and roost surveys should be conducted at other limestone hills (caves and rock shelters) in Lipis Geopark overall as these areas particularly Merapoh, may reveal more bat species records, collectively enhancing the bat conservation efforts in the country.

## Conclusions

Undoubtedly, the rich limestone karst landscape in Merapoh harbours a high number of bat species, with 865 individuals from 32 species recorded from eight Merapoh caves, totalling up to 38 species when combined from previous studies. Two caves are considererably notable: Gua Jinjang Pelamin harbouring the largest colony of *Eonycterisspelaea* found in Merapoh that provides essential pollination services here and Gua Gunting, the cave with the highest number of bat species recorded as of writing (19 species in total). Aside from the four new locality records for Pahang, this Merapoh bat species checklist contains several bat species that are of Least Concern for the global IUCN Red List, but their status in the country is Data Deficient. Such information is crucial in adding up to the whole picture of bat diversity knowledge in Malaysia. The capture of *Rhinolophusconvexus* at a lower elevation in Merapoh reflects the high potential of bat species diversity in this area, even more so with the inclusion of the Taman Negara Pahang Sungai Relau for future bat studies.

The significance of Merapoh within Lipis National Geopark as a bat conservation area is comparable to Krau Wildlife Reserve, Pahang, the third largest protected area in Peninsular Malaysia, which supports the highest bat species diversity recorded (69 species) from more than thirty years of bat research. Merapoh provides both permanent roosting structures (caves) and reliable foraging grounds for bats as the area is surrounded by Taman Negara and the various forest reserves, conducive for the long-term continuation of bat populations in Gua Jinjang Pelamin for *E.spelaea* and Gua Pasir Besar for *Miniopterusmedius* amidst anthropogenic disturbances like encroachment up to the limestone wall, irresponsible caving activities and forest fragmentation. Future bat research should be continued in Merapoh as there are still many limestone hills in Merapoh that have yet to be explored. Assessment of human disturbance in the caves and habitat enrichment near limestone hills should also be evaluated to create an integrated bat conservation management plan. Elements of bat diversity can also be integrated into the Merapoh ecotourism activity to educate the public on the importance of bats in the ecology of the tropical rainforest ecosystem. Lastly, this Merapoh bat species checklist can contribute to the country's bat conservation efforts and hopefully serve as a catalyst for others to conduct bat research here in Merapoh and Lipis Geopark overall.

## Supplementary Material

418B466E-5F1D-5314-A2DB-D2A1D6089CF2 10.3897/BDJ.12.e125875.suppl1 Supplementary material 1 Bats sampling data Merapoh in Lipis Geopark Data type Occurrence Brief description The occurrence data of all bats captured in all eight caves in Merapoh within the Lipis National Geopark, Pahang, Malaysia. File: oo_1060437.txt https://binary.pensoft.net/file/1060437 Aminuddin Baqi Hasrizal Fuad, Nur Zakirah Halmi, Jayaraj Vijaya Kumaran

## Figures and Tables

**Table 1.** GPS locations, number of trapping nights and trapping night dates at eight Merapoh caves.

No.	Caves	GPS location	Trapping nights	Date of sampling
1	Gua Air Mata Dayang	4°36'37.39"N, 101°59'37.0968"E	4	9/4/2021- 21/8/2021
2	Gua Persit	4°38'45.72"N, 102°00'23.38"E	4	23/7/2020-24/12/2020
3	Gua Gunting	4°42'31.94"N 101°59'11.88"E	8	7/9/2021-10/3/2022
4	Gua Jinjang Pelamin	4°37'16.03"N,102° 0'10.81"E	6	13/8/2020-16/10/2021
5	Gua Kalong	4°37'38.23"N, 101°59'36.15"E	4	3/3/2021-6/3/2021
6	Gua Katak	04°38'27.27"N, 102° 0'21.34"E	4	5/3/2022-11/3/2022
7	Gua Pasir Besar	4°34'9.90"N, 101°59'42.92"E	4	14/2/2021-17/2/2021
8	Gua Tahi Bintang	4°35'26.94"N, 101°59'19.59"E	5	5/3/2020-8/4/2021

**Table 2.** The bat species composition at the eight caves in Merapoh, Pahang.

No	**Species**	**GJP**	**GTB**	**GK**	**GAMD**	**GP**	**GG**	**GPB**	**GKt**
**Pteropodidae**									
1	*Cynopterusbrachyotis*	0	9	0	0	0	1	0	3
2	Cynopteruscf.brachyotis Forest	7	0	0	1	0	0	0	0
3	*Cynopterushorsfieldii*	2	0	1	2	3	1	0	5
4	*Cynopterussphinx*	0	0	0	0	0	2	0	2
5	*Eonycterisspelaea*	94	0	0	0	6	0	0	0
6	*Rousettusleschenaultii*	19	2	0	0	0	0	0	0
**Emballonuridae**									
7	*Emballonuramonticola*	0	0	1	0	0	14	0	0
**Megadermatidae**									
8	*Lyrodermalyra*	0	1	2	0	0	0	0	0
**Rhinolophidae**									
9	*Rhinolophusaffinis*	5	2	0	0	0	4	33	4
10	*Rhinolophuscoelophyllus*	0	0	0	0	0	1	0	0
11	*Rhinolophusconvexus*	0	0	0	0	0	1	0	0
12	*Rhinolophuspusillus*	9	3	6	1	0	4	15	2
13	*Rhinolophusrefulgens*	4	0	0	0	0	8	0	0
14	*Rhinolophusstheno*	1	12	4	0	0	1	55	3
**Hipposideridae**									
15	*Hipposiderosarmiger*	0	0	1	8	0	12	0	11
16	*Hipposiderosbicolor*	0	1	0	0	1	1	1	0
17	*Hipposideroscervinus*	3	0	0	0	0	0	3	0
18	*Hipposideroscineraceus*	0	0	1	4	4	15	0	1
19	*Hipposiderosdiadema*	5	0	0	0	0	0	0	0
20	*Hipposiderosdyacorum*	6	0	0	7	0	68	0	14
21	*Hipposiderosgaleritus*	0	0	0	0	0	0	1	1
22	*Hipposideroskunzi*	2	1	60	18	0	68	4	17
23	*Hipposideroslarvatus*	6	54	0	32	0	0	20	5
24	*Hipposideroslylei*	0	0	0	0	1	0	0	0
25	*Hipposiderospomona*	0	0	0	0	0	1	0	0
**Vespertilionidae**									
26	*Hesperoptenusblanfordi*	0	0	0	0	0	1	1	0
27	*Kerivoulapellucida*	1	0	0	0	0	0	0	0
28	*Kerivoulahardwickii*	0	0	0	0	0	1	0	1
29	*Murinasuilla*	0	0	0	0	0	1	0	0
30	*Myotisater*	0	0	0	1	0	0	0	0
**Miniopteridae**									
31	*Miniopterusmagnater*	0	0	0	0	0	0	1	0
32	*Miniopterusmedius*	0	1	0	1	0	0	41	0
	Total individuals (N)	164	86	76	75	15	205	175	69
	**Total number of species**	**14**	**10**	**8**	**10**	**5**	**19**	**11**	**13**
	Dominance (D')	0.112	0.556	0.701	0.275	0.4	0.274	0.206	0.177
	Evenness (J')	0.971	0.497	0.414	0.753	0.941	0.619	0.742	0.849
	Shannon-Wiener index (H')	2.235	0.967	0.666	1.565	1.034	1.675	1.78	1.954

**Keys; GJP**: Gua Jinjang Pelamin, **GTB**: Gua Tahi Bintang, **GK**: Gua Kalong, **GAMD**: Gua Air Mata Dayang, **GP**: Gua Persit (also known as Gua Baja), **GG**: Gua Gunting, **GPB**: Gua Pasir Besar, **GKt**: Gua Katak.

**Table 3.** Comparison of all bat diversity studies with their conservation status in Merapoh (PERHILITAN 2017, IUCN 2023).

No	Species	Ratnam et al. 1989	Bekong	This Study	IUCN Red List 2023	Red List Peninsular Malaysia 2017
	**Pteropodidae**					
1	*Balionycterisseimundi* Kloss, 1921	+	+	-	LC	LC
2	*Cynopterusbrachyotis* (Müller, 1838)	+	+	+	LC	LC
3	Cynopteruscf.brachyotis Forest	-	-	+	LC (grouped with *C.brachyotis*)	LC
4	*Cynopterushorsfieldii* Gray, 1843	+	-	+	LC	LC
5	*Cynopterussphinx* (Vahl, 1797)	-	+	+	LC	LC
6	*Eonycterisspelaea* (Dobson, 1871)	-	-	+	LC	NT
7	*Macroglossusminimus* (É. Geoffroy Saint-Hilaire, 1810)	-	+	-	LC	DD
8	*Rousettusleschenaultii* (Desmarest, 1820)*	-	-	+	NT	DD
	**Emballonuridae**					
9	*Emballonuramonticola* Temminck, 1838	-	-	+	LC	DD
	**Nycteridae**					
10	*Nycteristragata* (K. Andersen, 1912)	-	+	-	NT	LC
	**Megadermatidae**					
11	*Lyrodermalyra* (É. Geoffroy Saint-Hilaire, 1810)*	-	-	+	LC	DD
12	*Megadermaspasma* (Linnaeus, 1758)	+	-	-		
	**Rhinolophidae**					
13	*Rhinolophusaffinis* Horsfield, 1823	+	+	+	LC	LC
14	*Rhinolophuscoelophyllus* Peters, 1867*	-	-	+	LC	DD
15	*Rhinolophusconvexus* Csorba, 1997	-	-	+	DD	DD
16	*Rhinolophusluctus* Temminck, 1834	-	+	-	LC	LC
17	*Rhinolophuspusillus* Temminck, 1834	-	+	+	LC	DD
18	*Rhinolophusrefulgens* Andersen, 1906	-	-	+	LC (as *R.lepidus*)	DD
19	*Rhinolophusstheno* K. Andersen, 1905	-	-	+	LC	LC
	**Hipposideridae**					
20	*Hipposiderosarmiger* (Hodgson, 1835)	-	-	+	LC	LC
21	*Hipposiderosbicolor* (Temminck, 1834)	-	+	+	LC	LC
22	*Hipposideroscervinus* (Gould, 1854)	-	-	+	LC	LC
23	*Hipposideroscineraceus* Blyth, 1853	-	+	+	LC	LC
24	*Hipposiderosdiadema* (É. Geoffroy Saint-Hilaire, 1813)	+	-	+	LC	LC
25	*Hipposiderosdyacorum* Thomas, 1902	-	-	+	LC	DD
26	*Hipposiderosgaleritus* Cantor, 1846	-	-	+	LC	LC
27	*Hipposideroskunzi* Murray, Khan, Kingston, Akbar & Campbell, 2018	-	-	+	LC (as *H.atrox*)	VU
28	*Hipposideroslarvatus* (Horsfield, 1823)	-	+	+	LC	LC
29	*Hipposideroslylei* Thomas, 1913	-	-	+	LC	DD
30	*Hipposiderospomona* K. Andersen, 1918*	-	-	+	EN	DD
	**Vespertilionidae**					
31	*Hesperoptenusblanfordi* (Dobson, 1877)	-	-	+	LC	DD
32	*Kerivoulapellucida* (Waterhouse, 1845)	-	+	+	NT	LC
33	*Kerivoulahardwickii* (Horsfield, 1824)	-	-	+	LC	LC
34	*Murinasuilla* (Temminck, 1840)	-	+	+	LC	LC
35	*Myotisater* (Peters, 1866)	-	-	+	LC	DD
36	*Myotishorsfieldii* (Temminck, 1840)	+	-	-		
	**Miniopteridae**					
37	*Miniopterusmagnater* Sanborn, 1931	-	-	+	LC	Not assessed
38	*Miniopterusmedius* Thomas & Wroughton, 1909	-	-	+	LC	DD
	**Total number of species**	**7**	**13**	**34**		

**Keys**; + : Present, - : Absent, * : New locality record in Pahang.
